# Impact of artificial intelligence on the choice of radiology as a specialty by medical students from the city of São Paulo

**DOI:** 10.1590/0100-3984.2019.0101

**Published:** 2020

**Authors:** Gabriela Irene Garcia Brandes, Giuseppe D’Ippolito, Anderson Gusatti Azzolini, Gustavo Meirelles

**Affiliations:** 1 Escola Paulista de Medicina da Universidade Federal de São Paulo (EPM-Unifesp), São Paulo, SP, Brazil.; 2 Grupo Fleury, São Paulo, SP, Brazil.

**Keywords:** Artificial intelligence, Radiology, Medical students, Education, Specialty, Inteligência artificial, Radiologia, Estudantes de medicina, Educação, Especialidade médica

## Abstract

**Objective:**

To evaluate the impact of artificial intelligence (AI) on undergraduate medical students’ choice of radiology as a specialty.

**Materials and Methods:**

In February 2019, an anonymous online survey was sent to medical students. The research contemplated questions on how much students think they know about AI technologies, how much AI discourages them from choosing radiology as a specialty, and whether they believe there is a threat to the radiology job market.

**Results:**

A total of 101 students, most of them doing their internship, answered the questionnaire. More than half of them (52.5%) said they believe AI poses a threat to the radiology job market, but 64.3% claimed not to have proper knowledge about these new technologies, and 31.7% said they would like more information on the technologies’ operation and progress before making a decision on whether or not to practice radiology as a specialty.

**Conclusion:**

A significant proportion of the surveyed students perceive AI as a threat to the radiological practice, which impacts their career choice. However, the majority claims to have insufficient knowledge of it and believes more information is needed for decision-making.

## Abstract (pt)

**Objetivo:**

Avaliar o impacto da inteligência artificial (IA) na escolha de radiologia como especialidade médica por estudantes de medicina.

**Materiais e Métodos:**

Em fevereiro de 2019, uma pesquisa *online* anônima foi enviada a estudantes de medicina contemplando questões como: o quanto os alunos julgam conhecer as tecnologias de IA, o quanto estas os desestimulam a escolher radiologia como especialidade, e se julgam que o mercado de trabalho do radiologista está ameaçado.

**Resultados:**

Um total de 101 estudantes respondeu ao questionário, em sua maioria inserida no internato médico. Mais da metade dos alunos (52,5%) acreditava que o mercado de trabalho para o radiologista está ameaçado pela IA, mas 64,3% alegaram não ter muito conhecimento sobre essas novas tecnologias, e 31,7% gostariam de ter mais informações sobre o funcionamento e progresso da IA antes de tomar uma decisão sobre exercer ou não a radiologia como especialidade.

**Conclusão:**

Uma parte expressiva dos estudantes de medicina acredita que a IA é uma ameaça para a prática radiológica e esta percepção impacta sua escolha de carreira. No entanto, a maioria afirma ter um conhecimento insuficiente sobre o assunto e entende que é necessário um maior grau de informação para uma tomada de decisão.

## INTRODUCTION

Choosing a specialty is one of the crucial moments in the career of a medical student-a moment of doubt, reflection, and self-knowledge. For some, the choice is intuitive; for others, it needs to be well and carefully thought out, because it is not only affinity that plays a part in one’s choice of a career. The student takes several factors into account, such as the job market, desired lifestyle, field of study, and growth prospects for the specialty in the academy, among others^(1)^.

The link between the medical student and choice of radiology as a specialty has been discussed in the academic environment, mainly due to the development of new artificial intelligence (AI) technologies, which many consider potentially disruptive, to the point of changing the way we know and practice radiology^(2-5)^.

There are many questions confronting radiology with respect to its future, particularly for being at the technological forefront of medicine. Programs based on deep learning are the focus of several research projects worldwide^(3,6,7)^. The aim is to apply this technology to medicine, equipping computers to learn to recognize image patterns and improve the results from a database. Some even foresee that they will be able to diagnose diseases on their own in the near future. For example, a recent French study demonstrated an algorithm based on convolutional neural networks capable of detecting lesions of the meniscus by analyzing knee magnetic resonance imaging scans, presenting this algorithm as the first step toward the development of even more advanced AI diagnostic tools^(6)^.

A study recently conducted in Canada showed that anxiety related to AI’s impact on radiology discourages many medical students from considering it a career. The study suggests that the radiology community should educate students about AI’s potential repercussions to ensure that the specialty is perceived as a viable long-term career option^(2)^. On the other hand, a study on the subject conducted in Germany concluded that medical students are not worried about the possibility of AI replacing radiologists and are aware of its potential applications in and implications for the specialty and medicine. It suggests that radiology should lead students’ education in the context of these emerging technologies^(4)^.

Despite the relevance of the topic and the concerns it raises, few studies have sought to establish AI’s impact on the choice of medical specialty and, particularly, in the context of radiology.

Thus, considering this lack of adequate research, the importance of the subject, and the discrepant results of the few available studies^(2,4)^, the objective of the present study was to establish AI’s impact on the choice of radiology as a specialty by medical students in the city of São Paulo, Brazil.

## MATERIALS AND METHODS

A cross-sectional, optional, and anonymous survey was conducted online in February 2019-a Google Docs questionnaire was sent by e-mail to 360 randomly chosen students in different faculties of medicine in the city of São Paulo.

The questionnaire quizzed about aspects concerning individual perception of AI’s impact on radiology. Its main objective was to assess whether the development of AI technologies is discouraging the students in choosing radiology as a specialty.

The questionnaire comprised seven statements and a multiple-choice question on the theme. The statements were graded, according to the Likert scale, in levels of concordance ranging from 1 to 5: 1 I totally disagree; 2 I disagree; 3 I neither agree nor disagree; 4 I agree; 5 I totally agree. Answers 1 and 2 were considered negative, 3 indifferent, and 4 and 5 positive.

The statements included questions on how much the students think they understand AI technologies, how much such technologies discourage them from choosing radiology as a specialty, and whether they think there is a threat to the radiology job market.

According to the rules for the protection of human research subjects, studies based on anonymous and optional questionnaires do not require the approval of a research ethics committee^(8)^.

Statistical analysis of the information collected was performed in a descriptive manner, through the calculation of frequencies (in percentage).

## RESULTS

Responses of 101 students were collected through the survey. Most of them (60%) were in their sixth year, while 17% were in their fifth year and 23% in their fourth year. The survey did not include students in the first three years of graduation, as there is evidence in the literature that in the initial phase of medical education, students are yet to form an opinion on the specialty to follow^(1,9)^.

From the total, 53 (52.5%) said they perceive AI as a threat to the radiology job market and 37 (36.6%) said they believe it even threatens the radiologist’s assistance function. On the other hand, 65 (64.3%) claimed not to have much knowledge about new AI technologies and 32 (31.7%) said they would like more information on the functioning and development of these technologies before deciding whether to opt for radiology as a specialty.

Of all the students, 75 (74.3%) reported that they have never considered choosing radiology. Among the 26 (25.7%) who have, 18 no longer do-11 because of the development of AI technologies and 7 for reasons unrelated to AI. The complete results of the survey are presented in Table 1.

**Table 1:** Complete results of the questionnaire.

	1 I totally disagree	2 I disagree	3 I neither agree nor disagree	4 I agree	5 I totally agree
I am interested in taking up radiology as a specialty.	64 (63.3%)	19 (18.8%)	10 (9.9%)	4 (4.0%)	4 (4.0%)
I am interested in studying radiology to improve my clinical practice, without necessarily practicing it as my specialty.	2 (2.0%)	6 (5.9%)	13 (12.9%)	32 (31.7%)	48 (47.5%)
I have a lot of knowledge about new artificial intelligence technologies.	19 (18.8%)	46 (45.5%)	28 (27.7%)	5 (5.0%)	3 (3.0%)
I would like more information on the functioning and development of artificial intelligence technologies before making a decision on whether to opt for radiology as a specialty.	23 (22.8%)	21 (20.8%)	25 (24.7%)	10 (9.9%)	22 (21.8%)
The development of artificial intelligence technologies discourages the practice of radiology as a specialty.	27 (26.7%)	15 (14.9%)	31 (30.7%)	17 (16.8%)	11 (10.9%)
I believe artificial intelligence poses a threat to the radiology job market.	7 (6.9%)	16 (15.8%)	25 (24.8%)	31 (30.7%)	22 (21.8%)
I believe artificial intelligence poses a threat to the radiologist’s assistance function.	16 (15.8%)	22 (21.8%)	26 (25.7%)	26 (25.7%)	11 (10.9%)

## DISCUSSION

AI is the branch of science dedicated to the development of algorithms aimed at the execution of tasks traditionally associated with human intelligence, such as the ability to learn and solve problems; the term emerged for the first time in 1955^(5)^. AI is being widely discussed in our society, as is its impact on numerous daily and professional activities, which go beyond the scope of medicine and, more specifically, radiology^(5)^. Much of the fear this topic evokes is related to the limited understanding of most of those involved in these discussions. AI is a generic term that may encompass its lighter forms, such as an ordinary calculator that is only capable of performing the four basic mathematical operations, to its stronger forms, which may contemplate convolutional neuronal networks and, more specifically, deep learning and machine learning. Evidently, when assessing AI’s influence on radiological activity, the intended meaning relates to the stronger AI forms mentioned above^(3)^.

Although this topic is currently very important, few studies have sought to establish AI’s impact on some professions, including those linked to radiology. Our survey data suggest that medical students from the city of São Paulo have some concerns about the future of radiology, but most say they are unaware or know little about AI’s current level of evolution. This may be due to the fact that AI technologies are not actively addressed and discussed in medical school, i.e., the future residents of any specialty, in particular radiology, are not being taught about the upcoming opportunities and possibilities in this area of knowledge.

More than half of the respondents who had given up practicing radiology said they had changed their minds mainly because of AI’s increasing influence on clinical practice. However, it is interesting to note that this change in career direction is common, even outside the context of AI, and is observed in up to 70% of students until the end of the course, for various reasons^(1)^.

When discussing AI and radiology, important questions arise: can AI one day replace the radiologist’s function in image analysis? If yes, how long would it take and what then would be the radiologist’s role in the job market? Who would be responsible for an exam interpreted and reported by an algorithm? Answers to these and other questions are uncertain. It is not possible to accurately predict the future of a specialty in the long term, but much has been speculated on the subject.

In March 2019, the Radiological Society of North America (RSNA) published a report on the conclusions of its last meeting on AI and radiology. The RSNA provides numerous resources for research and education related to AI but recognizes that there is uncertainty about how to evaluate AI technology to ensure it is ready for clinical practice. It says there is ambiguity with respect to considering AI as an aid or competitor to the radiologist, and this can hinder the allocation of responsibility when there are undesirable clinical results arising from AI. The RSNA is willing to lead the assessment of the ethical and legal implications of AI in radiology, which is one of the priorities in its future initiatives^(10)^. Similarly, the Canadian Association of Radiologists believes radiologists should lead the discussion on the implementation and impact of AI in the field of imaging diagnosis^(5)^.

Despite all the concerns about the future, the specialty does not seem to be any less popular among students. In 2019, the residency test in radiology and diagnostic imaging at the Escola Paulista de Medicina of the Federal University of São Paulo had a sizeable list of candidates for its 12 vacancies, just as in 2015. However, according to the information gathered during the Congress of the American Association of University Radiologists, in Baltimore, April 2019, a survey by the American College of Radiology had found a 7% decrease in the demand for radiology residency programs in 2018 compared with the previous year; AI’s influence on this decrease is not yet clear.

Recent publications reveal that the determining factors in choosing a medical specialty depend on cultural values, demographic aspects, and healthcare and educational systems, varying greatly between different regions across the world^(1,9)^. In Brazil, the most important factors are affinity with the specialty, professional satisfaction, and lifestyle/quality of life related to the activity^(1,9)^. The valuation of these factors could justify, among medical students, the increased interest in radiology since the 1990s^(9,11)^.

Interestingly, the extracurricular contact during medical education is one of the factors considered relevant in the choosing of a specialty^(1,9)^. Thus, investing in interactions between students and radiology, providing the opportunity to better know and understand the profession’s main aspects and its integration with AI, could support a more conscious choice.

The distribution of medical specialties is an important factor to meet the needs of healthcare systems. In Brazil, there are 6.7 radiologists per 100,000 inhabitants, distributed unevenly across the national territory and varying widely among the Federation units-from 2.34:100,000 in Pará to 16.34:100,000 in the Federal District^(12)^. The density of radiologists in Brazil is lower than the average observed in the European Union (12.9:100,000) but higher than or close to that of some European countries, such as Italy (3.3:100,000) and the UK (7.5:100,000)^(12)^. In the last few years, there has been an increased interest in radiology, which has helped meet the demand for these professionals. On the other hand, the perception of an AI threat to the radiological career, reported by medical students, may inadvertently discourage them from choosing this specialty, leading to an undesirable demographic impact in the medium and long term.

Our study has some limitations, such as a restricted sample, as medical students only from one city in the country were evaluated. This limitation is also present in other studies with a similar goal^(1)^. Larger studies could confirm the results presented here. Also, the number of respondents was small (n = 101), but a response rate of around 28% was obtained, close to or higher than that of other studies with the same design^(1,2)^. Furthermore, we did not seek to establish differences between the participants on the basis of their gender, which influences the career choice^(9,11)^. Finally, the fact that we adopted a questionnaire with closed questions made it impossible to obtain complementary information of better quality or greater specificity to go deeper into the reasons that lead students to fear AI in clinical practice.

## CONCLUSION

The results of this study suggest that AI can have a negative impact on medical students’ choice of radiology as a specialty. On the other hand, there is evidence that indicates the existence of opportunities to minimize this trend by investing in education and quality information, clarifying realistically and objectively AI’s true role in the context of diagnostic imaging.
